# The Effect of Pre-Treatment (Blanching, Ultrasound and Freezing) on Quality of Freeze-Dried Red Beets

**DOI:** 10.3390/foods10010132

**Published:** 2021-01-10

**Authors:** Agnieszka Ciurzyńska, Julita Falacińska, Hanna Kowalska, Jolanta Kowalska, Sabina Galus, Agata Marzec, Ewa Domian

**Affiliations:** Department of Food Engineering and Process Management, Institute of Food Sciences, Warsaw University of Life Sciences, SGGW, 159c Nowoursynowska St., 02-776 Warsaw, Poland; julita.falacinska@gmail.com (J.F.); hanna_kowalska@sggw.edu.pl (H.K.); jolanta_kowalska@sggw.edu.pl (J.K.); sabina_galus@sggw.edu.pl (S.G.); agata_marzec@sggw.edu.pl (A.M.); ewa_domian@sggw.edu.pl (E.D.)

**Keywords:** red beet, freeze drying, blanching, freezing, ultrasounds

## Abstract

This paper presents the influence of blanching, ultrasonic processing and freezing conditions on selected physical properties of freeze-dried red beet, i.e., water activity, structure, porosity and shrinkage. Red beets subjected to a selected pre-treatment using its various parameters were frozen by three methods and then freeze-dried. Ultrasound reduced the water activity of samples. Blanching in water reduced shrinkage and improved porosity. In addition to the type of pre-treatment applied, the quality was also affected by freezing conditions before drying. Combined freezing resulted in the highest shrinkage and the lowest porosity and water activity. Slowly frozen samples were characterized by the best porosity.

## 1. Introduction

Red beet (*Beta vulgaris L.*) is a two-year vegetable valued for its high nutritional value. It owes its pro-health healing effect to macro- and microelements in the nutritional composition which result in the use of red beet for the prevention and treatment of many diseases [1]. Research confirms that beetroot juice contains more total phenols and flavonoids than citrus, cranberry and apples [2]. These vegetables have anti-cancer, antibacterial, anti-inflammatory and detoxifying properties. They improve the functioning of the cardiovascular and immune systems. They are also valued in treating skin problems, fighting fever and constipation. They are low in calories and easy to digest. They regulate cholesterol levels, lower blood pressure and improve liver function [1]. In recent years, dried vegetables have played an important role. They appear in the form of slices, flakes, cubes and ground powder. They may be consumed as healthy chips and snacks, but also as an ingredient in soups and seasonings [3]. The quality of dried food products depends on the pre-treatment used. Appropriate preliminary processes increase the drying efficiency and lead to an attractive final product. One of the most popular pre-treatment processes before drying is blanching (a short heat treatment consisting of quickly heating fruit or vegetables to a specific temperature and maintaining it for a specific time, followed by rapid cooling). The process inactivates enzymes, inhibits microbial growth and removes pesticide residues. Blanching also helps to peel fruit and vegetables, and is an important step before food canning, as it removes air from intercellular spaces. This pre-treatment process of degrading cellulose, rupturing cell membranes and changing the porosity of the cell wall increases the efficiency of bioactive compounds extraction [4,5] and reduces drying time [6]. Unfortunately, blanching in hot water causes the loss of valuable nutrients through leaching and high temperature [7]. Another problem is the water left after blanching. Such wastewater contains solids, e.g., pieces of vegetables, fruits, their seeds, large amounts of organic and inorganic compounds, e.g., sugars, proteins, pectins, which is why new blanching methods are currently being sought and developed. Innovative solutions include blanching by microwave, hot air with high humidity and infrared radiation in combination with hot air. Hybrid technologies combine several different techniques, e.g., traditional ultrasound blanching or vacuum blanching. Such combinations effectively deactivate enzymes while maintaining a high product quality and accelerating the heat transfer rate, thus reducing blanching time [5].

Ultrasound (US) creates elastic waves with a frequency between 20 kHz to 100 MHz, generated mechanically, thermally, optically and by using a reversible magnetic and electrical method [8]. Nowacka and Wendzik [9], on the basis of the literature, divided ultrasound according to the range of frequency into two groups: low intensity with low energy and high frequency (higher than 100 kHz), and high intensity with a high energy and low frequency between 20 and 100 kHz. The first type is considered as a non-destructive method, used to control the quality of food and to monitor the changes during the food production process. Whereas after high intensity ultrasound pre-treatment breaking cellular structures, activation and inhibition of chemical or physical alterations in food was found, which finally increased heat- and mass transfer-based processes. The authors argue that the scientific literature contains a lot of examples of the use of high ultrasound power in such food processes as drying and freezing. The use of ultrasound as a pre-treatment before drying food is not a new idea, but it has gained popularity in recent years [10,11]. This technique involves placing raw materials into a liquid medium, which is usually distilled water, and then applying an appropriate sonotrode connected to an ultrasound generator. This is one of the non-thermal techniques, which increases water diffusion during drying, reduces the drying time, resulting in significant cost savings [12]. Ultrasound has been applied prior to drying of, e.g., parsley leaves, bananas, mushrooms, brussels sprouts, cauliflower, carrot [13,14,15,16]. Ultrasound inactivates microorganisms and enzymes to preserve foodstuffs, especially when this technique is combined with heat techniques, since the low US process temperature protects thermolabile compounds [17]. Such pre-treatment before drying improves the drying kinetics and reduces the energy costs [16]. This is a promising technique which could be used for food pre-treatment [9].

Freeze-drying involves the removal of water by the sublimation of frozen material, i.e., the transition of the solvent into vapor without the liquid state. The freeze-drying process consists of three stages: freezing the material, ice sublimation and desorption. The quality of the product depends to a large extent on the first stage of the process, i.e., the proper freezing of the material. The resulting ice crystals consist of up to 99% of solid water. Their size has a significant impact on the porosity and structure of the dried material. Shock freezing promotes the formation of a large number of small crystals, resulting in the reduction of cell damage while slow freezing creates large crystals, which means that the product can be dried faster due to more intensive water vapour movement [18].

The aim of this work was to determine the impact of the type and pre-treatment parameters on the quality of freeze-dried red beet. The scope of this work included preparation of the raw material, assessment of the impact of blanching, freezing and ultrasound processing on selected quality indicators of freeze-dried red beets.

## 2. Materials and Methods

### 2.1. Material

Red beets (*Beta vulgaris*) (0) purchased at a local supermarket in Warsaw (Poland) and stored in a temperature range 4–6 °C were used. The whole tubers (20 pcs, diameter about 15 cm) were cut into 5-mm-thick slices using a slicer, and then 20-mm-diameter cylinders were cut. The sample names are shown in Table 1.

### 2.2. Technological Methods

#### 2.2.1. Blanching

The samples were blanched in distilled water at 65 °C for 10 and 15 min, and at 85 °C for 1 and 5 min, and then cooled using a sieve under cold running water for 1 min, and dried on filter paper. The samples were prepared in triplicate.

#### 2.2.2. Ultrasonic Pre-Treatment

The red beet samples were placed in a ultrasonic bath (MKD-3, MKD Ultrasonics, Stary Konik, Poland, internal dimensions: 240 × 140 × 110 mm) at frequency of 40 kHz, at total power of sonotrodes of 180 W for 5 and 10 min. The ratio of raw material to water was 1:4. This pre-treatment was conducted at room temperature (23 ± 1 °C, microprocessor temperature controller). Next, the samples were dried on filter paper. Samples were prepared in triplicate.

#### 2.2.3. Freezing

Before freeze-drying, the pre-treated raw material was frozen by three methods:

− fast freezing—in the ProfiMaster Personal Freezers PMU series 0380 (National Lab GmbH, Schleswig-Holstein, Germany) shock freezer, using a low freezing temperature of −80 °C for 2 h,
− slow freezing—in a chamber freezer (Whirlpool, Siena, Italy) at −18 °C, with storage for 2 weeks,
− combined freezing—using a low freezing temperature in a shock freezer −80 °C for 2 h and storage of samples for 2 weeks in a chamber freezer at −18 °C.

The control sample was frozen with each of the three methods, but without blanching and ultrasound. Samples were prepared in triplicate.

#### 2.2.4. Freeze-Drying

Freeze drying was carried out in ALPHA 1–4 freeze dryer (Christ, Osterode am HarzGermany) for 48 h at a heating shelf temperature of 10 °C, a constant pressure of 63 Pa and a safety pressure of 103 Pa. During the 48 h of the freeze-drying process, the temperature of the representative samples was controlled and drying was conducted until the temperature in the center of samples was about 10 °C. That indicates that all ice crystals were sublimated. Dried samples were stored in glass vessels and sealed at room temperature until investigations. Samples were prepared in triplicate.

### 2.3. Analytical Methods

#### 2.3.1. Water Activity Determination

Water activity was determined in three repetitions for every sample, at a temperature of 25 ± 1 °C, with the use of HygroLab C1 ROTRONIC (Rotronic AG, Bassersdorf, Germany) according to the manufacturer’s instructions. One sample of the tested material was placed into the four chambers of the apparatus and the chambers were tightly closed. After about 5 min, the results were determined. From the 4 results obtained, 3 repeatable ones were selected. The apparatus can measure both water activity and equilibrium relative humidity. The minimized internal volume of the sensor chamber allows for a fast equilibrium, and temperature stability during measurements is ensured by metal construction.

#### 2.3.2. The Shrinkage Determination 

Determination of the shrinkage was carried out using the buoyancy method in triplicate. For the samples, before drying, distilled water was used. To determine the volume of the dried samples, water was replaced with sea sand to avoid water absorption by the freeze-dried material [19]. Based on the change in volume of material, the shrinkage was calculated:

(1) $$S=1-\;\frac{V_{d}}{V_{o}}*100\;$$

where

*V_d_*—the volume of dried sample
*V_o_*—the volume of raw sample

#### 2.3.3. The Porosity Determination 

The porosity was measured using a Quantachrome Stereopycnometer helium pycnometer in triplicate according to the manufacturer’s instructions [20].

##### Porosity

(2) $$P_{c}=\frac{d-d_{p}}{d}*100$$

where

*P_c_*—whole porosity [%]
*d*—apparent density of the material measured by pycnometer [g/cm^3^]
*d_p_*—real density [g/cm^3^]

#### 2.3.4. Structure

The internal structure of the dried samples was determined on the basis of photos taken with a TM-3000 scanning microscope (HITACHI, Japan) at 100 times magnification (in Supplementary Materials). A piece about 2 mm thick was cut with a razor blade from the inner surface of the dried red beet, which was then coated with gold.

### 2.4. Statistical Methods

The results obtained were subjected to a statistical analysis using the STATGRAPHICS Plus 4.1 software (Statgraphics Technologies, Inc., The Plains, VA, USA), using one-way analysis of variance and comparing the means using a student’s *t* test at a significance level of α = 0.05. The other parameters were determined using MS Excel 2007.

## 3. Results

### 3.1. The Effect of Type and Parameters of Pre-Treatment on the Quality of Freeze-Dried Red Beet

The impact of pre-treatment conditions and parameters on the selected quality indicators of red beet was assessed. Samples without pre-treatment (control) were compared with samples blanched in water at 85 °C for 1 and 5 min and in water at 65 °C for 10 and 15 min, and also sonicated for 5 and 10 min (Table 1). The freezing and freeze-drying stages were the same for each test. A statistical analysis was carried out only for dried material.

#### 3.1.1. Water Activity

It was shown that the water activity (a_w_) of raw red beet was 0.975.

- Quick freezing

**Figure 1:**
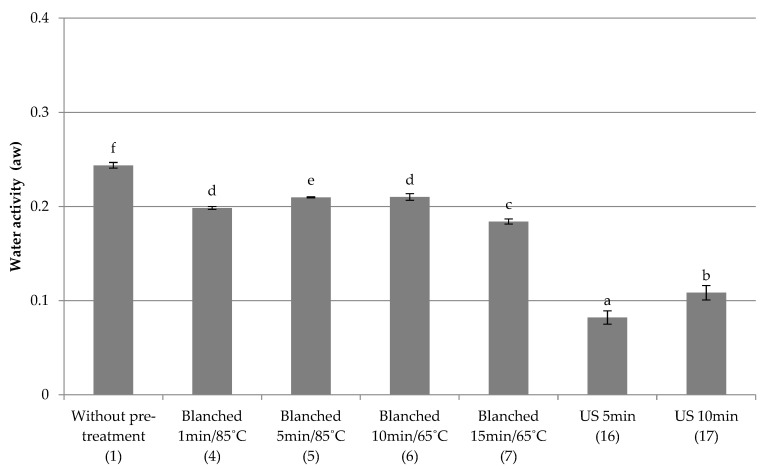
The effect of the type and parameters of pre-treatment on the water activity of freeze-dried beetroot frozen with the quick method. Designation in Table 1. a–f—the same letters in each figure indicate homogeneous groups at *p* < 0.05 levels.

The influence of the applied pre-treatment and quick freezing on water activity was investigated. The results are shown in Figure 1. The control sample (1) was red beet not subjected to any pre-treatment, frozen at −80 °C for 2 h. Its water activity was 0.24 and was the highest value the freeze-dried samples. The use of blanching (4–7) increases the water activity of samples in comparison to those subjected to ultrasound treatment (16–17), and lowers the tested parameter compared to the control sample (1). The lowest water activity was found for samples subjected to ultrasound (16–17). Increasing the duration of ultrasonic treatment (16–17) increased a_w_ from 0.08 to 0.11. The statistical analysis showed that the samples blanched for 1 min at 85 °C (4) obtained similar values of water activity as the samples blanched during 10 min at 65 °C (6). The remaining samples differed in a statistically significant way.

- Combined freezing

**Figure 2:**
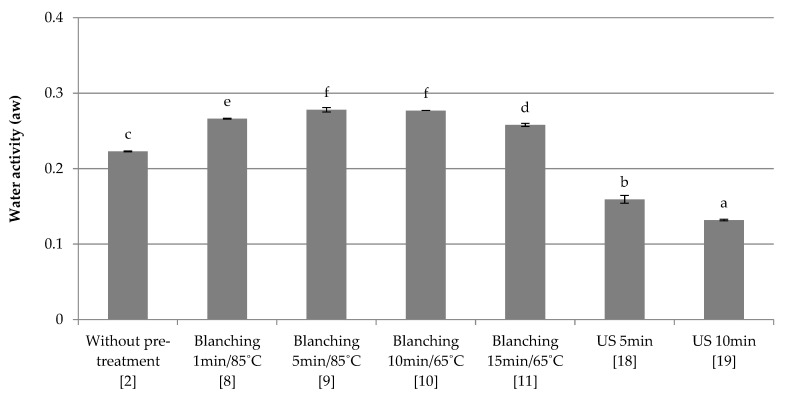
The effect of the type and parameters of pre-treatment on the water activity of freeze-dried beetroot frozen with the combined method. Designation in Table 1. a–f—the same letters in each figure indicate homogeneous groups at *p* < 0.05 levels.

**Figure 3:**
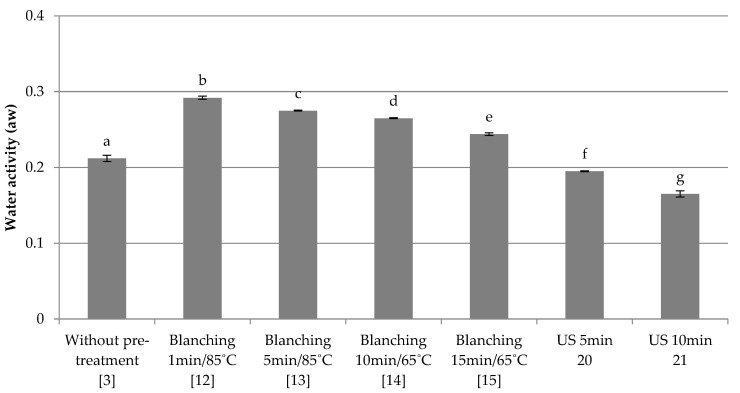
The effect of the type and parameters of pre-treatment on the water activity of freeze-dried beetroot frozen with the slow method. Designation in Table 1. a–g—the same letters in each figure indicate homogeneous groups at *p* < 0.05 levels.

The influence of the applied pre-treatment and combined freezing on water activity was investigated. The results are shown in Figure 2. The control sample (2) was red beet without any pretreatment, frozen by a combined method, and its water activity was 0.22. Red beet blanching (8–11) increases the water activity compared to other freeze-dried samples. Increasing the blanching time at 85 °C (8–9) increases this parameter, and decreases it at 65 °C (10–11). Samples 9 and 10 did not differ in a statistically significant way. As in the case of quick freezing (Figure 1) and slow freezing (Figure 3), samples subjected to ultrasound waves (18–19) showed the lowest water activity. However, a_w_ decreased with increasing ultrasound duration. The performed statistical analysis showed a significant influence of the type and parameters of the applied pre-treatment on water activity.

- Slow freezing

The influence of the applied pre-treatment and slow freezing on water activity was investigated. The results are shown in Figure 3. The control sample (3) was red beet without any pretreatment, frozen by the slow method, and its water activity was 0.21. As in the case of combined freezing, the samples subjected to blanching showed the highest water activity among freeze-dried products. The highest value was obtained for the sample blanched for 1 min at 85 °C and frozen at −18 °C (12). The high temperature of blanching, which significantly changed the structure and the method of freezing itself, may have contributed to this. With the extension of the blanching time, the water activity of the dried fruit decreased (12–15). The lowest values of water activity were found for samples subjected to ultrasound treatment (20–21), especially when the treatment time was 10 min (21). Ultrasound reduced water activity in comparison to the control sample (3). The performed statistical analysis showed significant differences between the tested samples.

#### 3.1.2. Porosity

- Quick freezing

**Figure 4:**
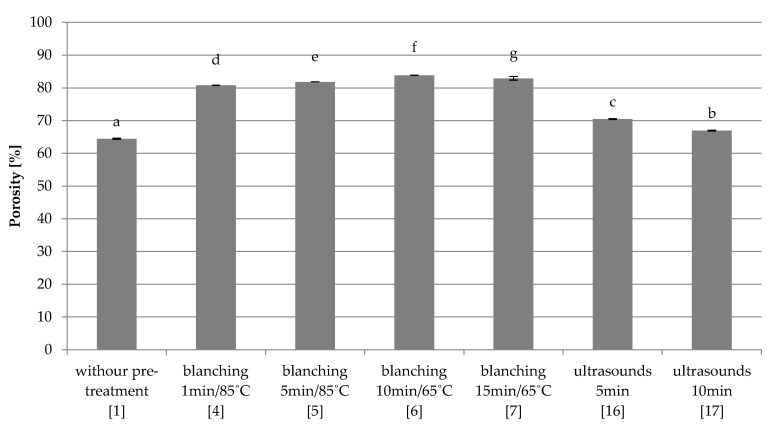
The effect of the type and parameters of pre-treatment on the porosity of freeze-dried beetroot frozen with the quick method. Designation in Table 1. a–g—the same letters in each figure indicate homogeneous groups at *p* < 0.05 levels.

The lack of pre-treatment in the control sample (1) resulted in the lowest porosity of 64.5% (Figure 4). Red beet samples subjected to blanching in water have the highest porosity at the level of about 83% (4–7). Increasing the duration of treatment significantly changed the porosity of the dried material. Ultrasonic treatment (16–17) significantly increased the porosity of the dried material compared to the control sample (1), but the extended time of ultrasound treatment reduced it for ~3%.

- Combined freezing

**Figure 5:**
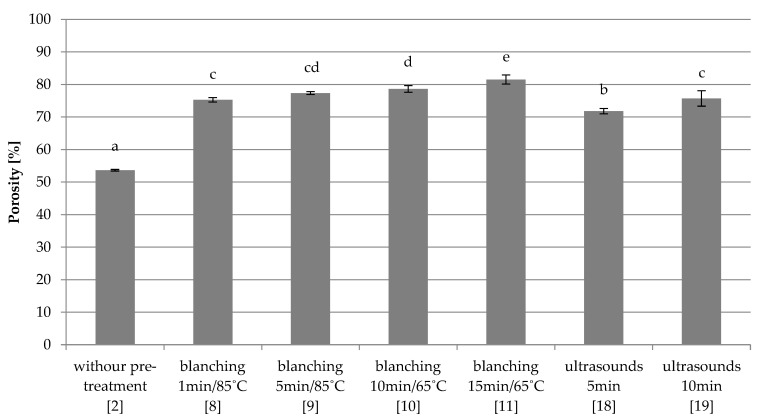
The effect of the type and parameters of pre-treatment on the porosity of freeze-dried beetroot frozen with the combined method. Designation in Table 1. a–e—the same letters in each figure indicate homogeneous groups at *p* < 0.05 levels.

Freeze-dried red beet without any pre-treatment (2) had the lowest porosity (53.6%) (Figure 5), similarly to the samples frozen by the fast method (Figure 4). Samples subjected to blanching in water (8–11) were characterized by the largest porosity of about 78%, and increasing the blanching time caused an increase of this parameter. The use of ultrasound (18–19) also significant increased the porosity of dried samples compared to the control sample (2), and the extension of the treatment time increased the porosity from 71.8% to 75.7%.

- Slow freezing

**Figure 6:**
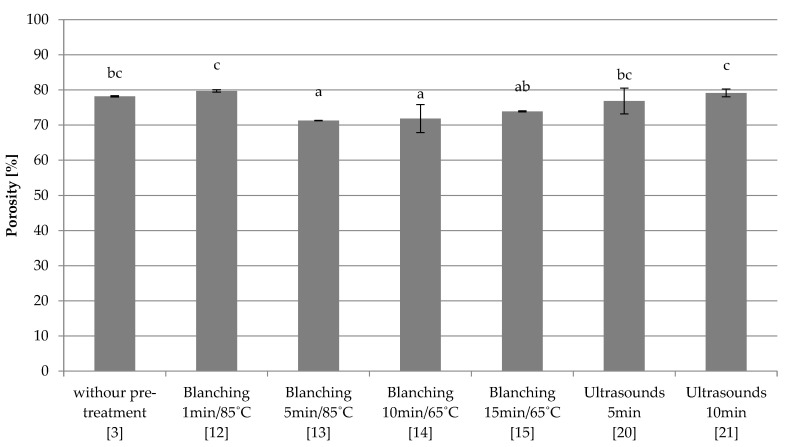
The effect of the type and parameters of pre-treatment on the porosity of freeze-dried beetroot frozen with the slow method. Designation in Table 1. a–c—the same letters in each figure indicate homogeneous groups at *p* < 0.05 levels.

The porosity of the freeze-dried control sample (3) without pre-treatment, frozen slowly, was 78.2% (Figure 6). Blanching in water in most cases (13–15) caused a statistically significant decrease of this parameter in comparison to the control sample (3), and the blanching time was insignificant. Only sample 12 did not differ significantly from the control sample (3). The samples subjected to ultrasonic treatment (20–21) showed similar porosity to the sample without pre-treatment (3), and the prolonged use of ultrasound caused a slight increase in porosity from 76.9% to 79.14%.

#### 3.1.3. Shrinkage

- Quick freezing

**Figure 7:**
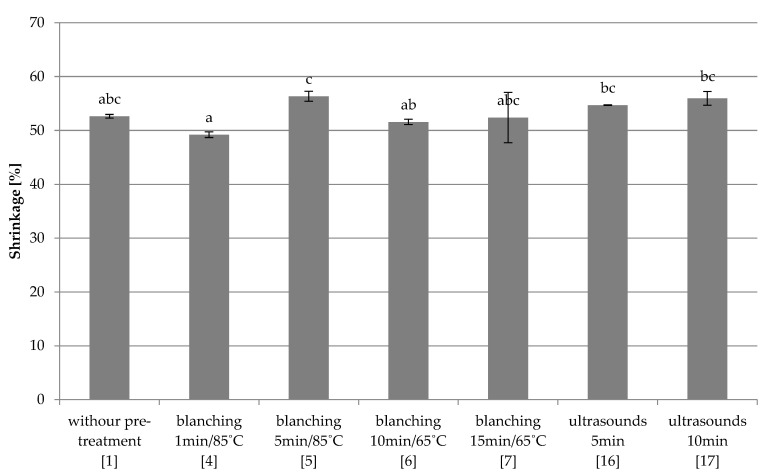
The effect of the type and parameters of pre-treatment on the shrinkage of freeze-dried beetroot frozen with the quick method. Designation in Table 1. a–c—the same letters in each figure indicate homogeneous groups at *p* < 0.05 levels.

It was shown that the use of pre-treatment in hot water or by ultrasound (4–7, 16–17) does not cause statistically significant changes in shrinkage compared to the control sample (1) (Figure 7). Increasing the blanching time only at 85 °C (5) resulted in a statistically significant increase in shrinkage compared to sample 4.

- Combined freezing

**Figure 8:**
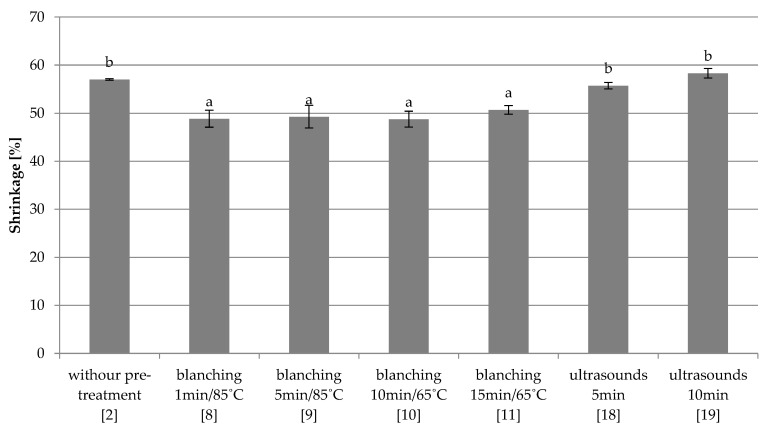
The effect of the type and parameters of pre-treatment on the shrinkage of freeze-dried beetroot frozen with the combined method. a,b—the same letters in each figure indicate homogeneous groups at *p* < 0.05 levels.

It has been shown that the use of blanching in water (8–11) causes a statistically significant reduction of samples’ shrinkage in relation to the control sample (2) (Figure 8). No significant influence of blanching conditions on the examined parameter was found. The use of ultrasonic treatment (18–19) did not significantly change the shrinkage of the red beet compared to the control sample (2), but the shrinkage was significantly higher compared to the samples blanched in water. Similarly to water blanching, the ultrasonic treatment conditions were statistically insignificant.

- Slow freezing

**Figure 9:**
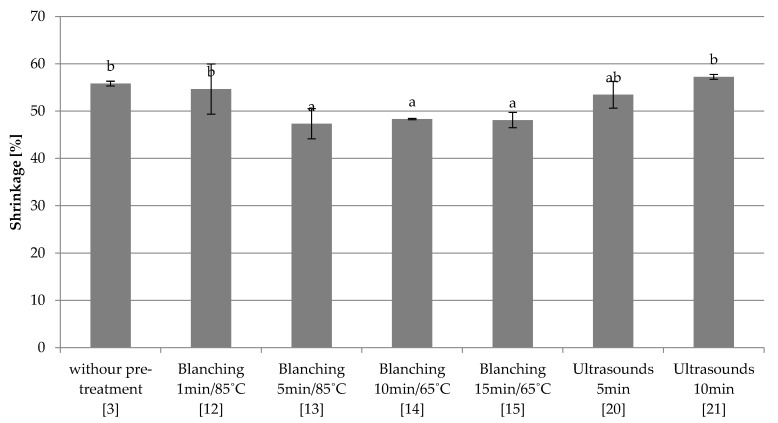
The effect of the type and parameters of pre-treatment on the shrinkage of freeze-dried beetroot frozen with the slow method. Designation in Table 1. a,b—the same letters in each figure indicate homogeneous groups at *p* < 0.05 levels.

The control sample (3) frozen at −18 °C and subjected to freeze-drying was characterized by a shrinkage of 55.82% and was similar to a sample blanched by 1 min in water at 85 °C (12) and samples subjected to ultrasound (20–21) (Figure 9). Similarly to fast and combined freezing, also in this case, blanching in water (13–15) resulted in a significant reduction of shrinkage of most samples. Extending the blanching time only at 85 °C (12–13) significantly reduced the shrinkage of the material. Ultrasonic treatment (20–21), as in the case of combined freezing, did not cause significant changes in shrinkage in relation to the control sample, and the process conditions were insignificant.

### 3.2. The Effect of Freezing Conditions on the Quality of Freeze-Dried Red Beet

The analysis of the impact of freezing conditions before the freeze-drying process on the quality of dried samples was carried out by comparing red beets frozen at −80 °C for 2 h (quick freezing), −80 °C for 2 h, and then stored for 2 weeks at −18 °C (combined freezing) and frozen at −18 °C for 2 weeks (freezing free). The parameters of the freeze-drying process were the same for each test.

#### 3.2.1. Water Activity

The effect of freezing conditions on the water activity of freeze-dried red beet was investigated (Table 2). The statistical analysis showed a significant impact of freezing conditions on water activity. The quick-frozen red beet (1) was characterized by the highest water activity, and the sample with the lowest activity was initially frozen for 2 h at the temperature of −80 °C, and then stored for 2 weeks at −18 °C (2).

#### 3.2.2. Porosity

**Figure 10:**
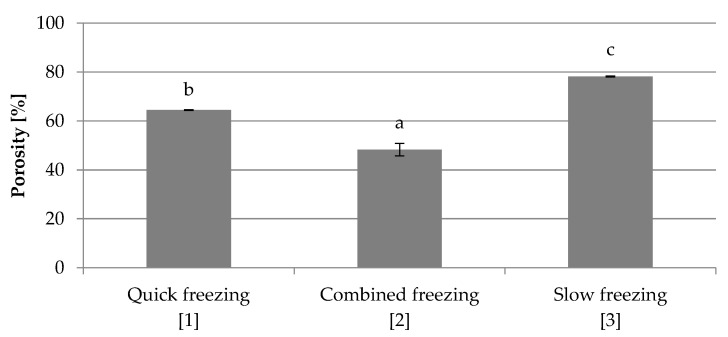
The effect of freezing conditions on the porosity of freeze-dried beetroot. Designation in Table 1. a–c—the same letters in each figure indicate homogeneous groups at *p* < 0.05 levels.

**Figure 11:**
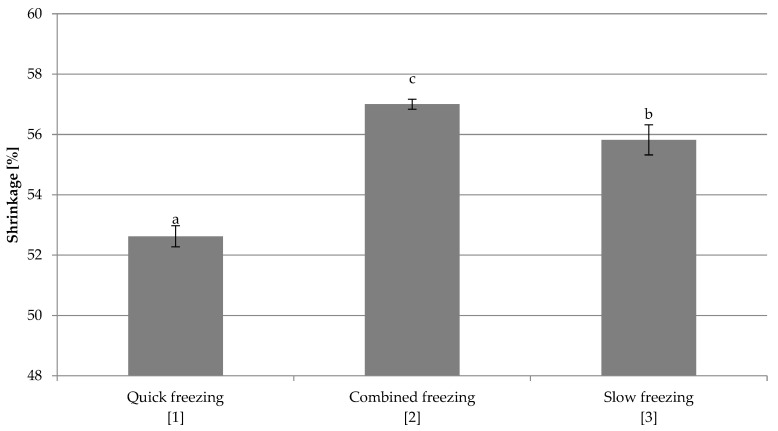
The effect of freezing conditions on the shrinkage of freeze-dried beetroot. Designation in Table 1. a–c—the same letters in each figure indicate homogeneous groups at *p* < 0.05 levels.

The porosity results (Figure 10) confirm the shrinkage results (Figure 11). It has been shown that the slow-frozen samples have the highest porosity (3), and the lowest porosity is obtained after using the combined freezing method (2). These differences were caused by the damage that occurred inside the material due to the use of different freezing conditions. The statistical analysis showed that differences were significant.

#### 3.2.3. Shrinkage

It was shown that the freezing method has a significant impact on the shrinkage of freeze-dried red beet (Figure 11). The smallest shrinkage occurred in quick frozen samples (1), and the largest in samples frozen by the combined method (2). Samples frozen at −18 °C showed highest shrinkage, most likely due to the destruction of the internal structure by formation of larger ice crystals. Quick freezing caused the formation of small ice crystals; there was no destruction of the structure inside the material, so the red beet shrinkage was smaller.

## 4. Discussion

It was shown that blanching increases the water activity of samples in comparison to those subjected to ultrasound treatment, and decreases this parameter compared to the control sample. The lowest water activity was found for samples subjected to ultrasound. The reason may be the cavitation phenomenon occurring during this treatment, which contributed to the increase in temperature and pressure, thus causing damage to the structure and resulting in more effective removal of water from the material [21]. Fernandes et al. [22] explained that micro-channels are created during sonication, and water may be easier removed from material.

Similar results were obtained by Deng and Zao [23], who compared the effect of drainage with periodic pressure reduction and the use of ultrasound on the quality of freeze-dried apples. They showed that ultrasonically treated samples have lower water activity than vacuum treated ones. Moreover, Ciurzyńska et al. [24] found that the use of ultrasound during dehydration reduces the water activity of some dried fruit compared to other samples. Cao et al. [25] investigated that barley grass pre-treated with ultrasounds before freeze-drying obtained lower water activity and with elongation of sonification time water activity decreased. Some authors claimed that strongly attached moisture may be removed by cavitation which occurs during ultrasound pre-treatment. Microscopic channels are created which reduce the diffusion boundary layer and increase the convective mass transfer in the fruit tissue [26]. An increase of water activity for samples frozen using the quick method and pre-treated longer with ultrasounds may be connected with the different structures of samples (Figure S1—supplementary materials). This indicates that freezing conditions are important for properties of samples sonificated at different times. In the other freezing methods, samples conducted by ultrasonic pre-treatment obtained similar structures for 5 and 10 min. During blanching, the samples absorbed water, which may explain the higher water activity of the blanched red beet. Similar results were obtained by Ciurzyńska et al. [27], which examined the properties of freeze-dried pumpkin.

Thermal processes cause numerous changes in the cellular structure of the product, including changes in the shape and size of cells. Thermal stress damages the internal structure, causing cracks, gaps and voids [28]. In visual assessment, pre-blanched red beets were characterized by larger pores compared to the other samples (Figures S1–S3—supplementary materials). In addition, Canet et al. [29] which examined the influence of blanching parameters on the quality of dried green beans, highlights the destruction of the structure caused by pre-treatment, and as the time was extended and the temperature of the process increased, cell walls were more degraded. Moreover, Nowacka et al. [30] have shown that during the blanching process, cell thermal disruption occurred in cranberries, and many small pores were visible on SEM images. Liu and Scanlon [31] have shown that blanching time has little effect on the potato strips structure, whereas the increase of temperature and time softened it. Samples treated by ultrasound in visual assessment are characterized by very small, partially open pores. Structure observations were confirmed by porosity measurement. Red beet samples subjected to blanching in water had the highest porosity, and increasing the blanching time caused an increase of this parameter. In addition, Wang et al. [32] found an increase of porosity of blanched and freeze-dried apple slices in comparison with the control sample. The use of ultrasound also resulted in an increase in dried material porosity compared to the control sample, and the extension of the treatment time increased this parameter too. Fijałkowska et al. [33] obtained similar results for dried apples pre-treated by ultrasound and showed that dried tissue subjected to ultrasound treatment was characterized by a lower density and a more porous structure. The authors also could not clearly determine the effect of ultrasound treatment time on microstructure changes. The fact that the water blanching or ultrasonic pre-treatment did not cause changes in shrinkage compared to the control sample was unexpected. The analysis of the results suggests that the determination methodology may be affected by error [19], since freeze-dried samples are usually characterized by shrinkage up to 15–20%. Similar shrinkage values were obtained by Koc et al. [34] for quince. Akonor and Tortoise [35] also showed that the shrinkage of chayote was more pronounced when blanching was applied, compared to the control samples. The presented work showed that extending the blanching time at only 85 °C significantly reduced the shrinkage of the material. Moreover Kapadiya et al. [36] obtained shrinkage decreases for dried potato slices with the increases of temperature and blanching time. However, Nowacka et al. [37] have shown that the volume of cranberries did not change after the ultrasonic treatment.

The investigations of freezing conditions on the water activity of freeze-dried red beet have shown that the quick-frozen sample was characterized by the highest water activity, and the lowest value was obtained for frozen by the combined method. This may be due to the fact that most of the water was frozen. Similar research results were obtained by Ciurzyńska et al. [27] while comparing the effect of the freezing method on the properties of freeze-dried pumpkin. Freezing conditions change the internal structure of the material and the smallest and most numerous pores were obtained in the quickly frozen sample. Freezing by the combined method created numerous closed pores, while slow freezing damaged the structure of dried red beet. Large ice crystals were formed in cell spaces, causing greater damage compared to other freezing methods (Figure S4—supplementary materials). Ceballos et al. [18] confirmed that faster freezing rates produced a decrease in the pore size of the final micro-structure of the freeze-dried soursop fruit pulp. Similar results were obtained by Wang et al. [32] for apple slices. Structure observations were confirmed by shrinkage and porosity tests. Quick frozen sample was characterized by the smallest shrinkage, and the largest shrinkage was observed for the sample frozen by the combined method. The quick freezing caused the formation of small ice crystals and there was no damage to the internal structure of the material so the red beet shrinkage was smaller. Similar results were obtained by Ciurzyńska et al. [27], who showed that the pumpkin subjected to rapid freezing was characterized by the smallest shrinkage. The shrinkage results obtained by using this method are higher than expected. Usually, freeze-dried samples show shrinkage of up to 20% [38], which may confirm the assumptions about the imperfections of the applied methodology.

## 5. Conclusions

This study examined the impact of the type and pre-treatment parameters on the selected properties of freeze-dried red beet. The findings are as follows:

1. The quality and properties of freeze-dried red beets depended on the type of pre-treatment used. However, due to the varied results, it was difficult to clearly determine which parameters were the most and which least favorable.

2. Ultrasonic treatment resulted in obtaining the dried material with the lowest water activity, and increasing its duration contributed to obtaining even lower values of the tested parameter. Among the blanched samples, the highest values were obtained for samples blanched at a high temperature of 85 °C.

3. Ultrasound compared to blanching in water caused higher shrinkage of the material. However, in both cases usually, the change in parameters did not contribute to changes in contraction. In most cases, blanching had an influence on shrinkage decrease compared to the control sample, whereas ultrasounds did not.

4. In most cases both, the type of pre-treatment and the change in its parameters affected the porosity of the red beet. Blanching in water at 65 °C for 15 min both in fast and combined freezing was the most beneficial method and resulted in the highest porosity of the dried material. In both cases of freezing, ultrasonically treated samples had the worst results, but a higher porosity than samples without pre-treatment.

5. Freezing conditions affected the red beet’s water activity. Nevertheless, that fast freezing resulted in the highest water activity, and the use of combined freezing contributed to the lowest value of this parameter. For the food industry, this may be not important because all the results were low and similar.

The shrinkage was also on a similar level, but red beet subjected to combined freezing was characterized by the greatest shrinkage, and thus the smallest porosity. Differences in porosity were larger. Based on the results obtained, it can be concluded that the most advantageous method turned out to be quick freezing, which resulted in the freeze-dried samples with the lowest shrinkage and best porosity.

## Figures and Tables

**Table 1:** Sample code of freeze-dried red beet.

Sample Code	Pre-Treatment	Freezing [Temp/Time]	
(1)	-	−80 °C/2 h	quick freezing
(2)	-	−80 °C/2 h, storage—18 °C, 14 days	combined freezing
(3)	-	−18 °C/14 days	slow freezing
(4)	1 min, 85 °C, H_2_O	−80 °C/2 h	quick freezing
(5)	5 min, 85 °C, H_2_O	−80 °C/2 h	
(6)	10 min, 65 °C, H_2_O	−80 °C/2 h	
(7)	15 min, 65 °C, H_2_O	−80 °C/2 h	
(8)	1 min, 85 °C, H_2_O	−80 °C/2 h, storage—18 °C/14 days	combined freezing
(9)	5 min, 85 °C, H_2_O	−80 °C/2 h, storage—18 °C/14 days	
(10)	10 min, 65 °C, H_2_O	−80 °C/2 h, storage—18 °C/14 days	
(11)	15 min, 65 °C, H_2_O	−80 °C/2 h, storage—18 °C/14 days	
(12)	1 min, 85 °C, H_2_O	−18 °C/14 days	slow freezing
(13)	5 min, 85 °C, H_2_O	−18 °C/14 days	
(14)	10 min, 65 °C, H_2_O	−18 °C/14 days	
(15)	15 min, 65 °C, H_2_O	−18 °C/14 days	
(16)	5 min, 40 kHz/180 W	−80 °C/2 h	quick freezing
(17)	10 min, 40 kHz/180 W	−80 °C/2 h	
(18)	5 min, 40 kHz/180 W	−80 °C/2 h, storage—18 °C/14 days	combined freezing
(19)	10 min, 40 kHz/180 W	−80 °C/2 h, storage—18 °C/14 days	
(20)	5 min, 40 kHz/180 W	−18 °C/14 days	slow freezing
(21)	10 min, 40 kHz/180 W	−18 °C/14 days	

**Table 2:** The effect of freezing conditions on the water activity of freeze-dried beetroot. Designation in Table 1. a–c—the same letters in in the same column indicate homogeneous groups at *p* < 0.05 levels.

Sample Name	Water Activity ± Standard Deviation
(1)	^a^ 0.243 ± 0.003055
(3)	^b^ 0.223 ± 0.000577
(2)	^c^ 0.211 ± 0.004041

## Data Availability

Data of investigations are available from the authors.

## Supplementary Materials

The effect of type and parameters of pre-treatment on the structure of freeze-dried red beet. The following are available online at https://www.mdpi.com/2304-8158/10/1/132/s1, Figure S1: Beetroot frozen by the quick method, Figure S2: Beetroot frozen by the combined method, Figure S3: Beetroot frozen by the slow method, Figure S4: The effect of freezing conditions on the structure of freeze-dried beetroot.
