# Supplementary material for: The Effect of Pre-Treatment (Blanching, Ultrasound and Freezing) on Quality of Freeze-Dried Red Beets
Source: Foods. 2021 Jan 10;10(1):132. doi: 10.3390/foods10010132 (PMC7827667; doi:10.3390/foods10010132)
Supplement: Supplementary file 1 [file foods-10-00132-s001.pdf]

The Effect of Type and Parameters of Pre-Treatment on the Structure of Freeze-Dried Red Beet

Using the scanning microscope, the internal structure of the freeze-dried red beet subjected to various pre-treatments was observed, compared to the control sample, which was red beet pre-frozen and then freeze-dried.

Quick Freezing

The control sample (1) was freeze-dried red beet subjected to rapid freezing at −80 ˚C. Its structure is characterized by small, partially open pores of similar size, evenly distributed. In visual assessment, pre-blanched samples (4–7) have larger pores compared to the control sample (1) and ultrasound red beet (16–17) (Figure S1). Such observations confirm the results of porosity (Figure 4. publication, main text) Between samples 4-7 the differences are not visible in the visual assessment. The structure is not uniform and the pores are large and partly open. Samples exposed to ultrasound (16–17) in visual assessment are characterized by very small, partly open pores. The irregular shape of the pores makes it impossible to make geometric measurements.

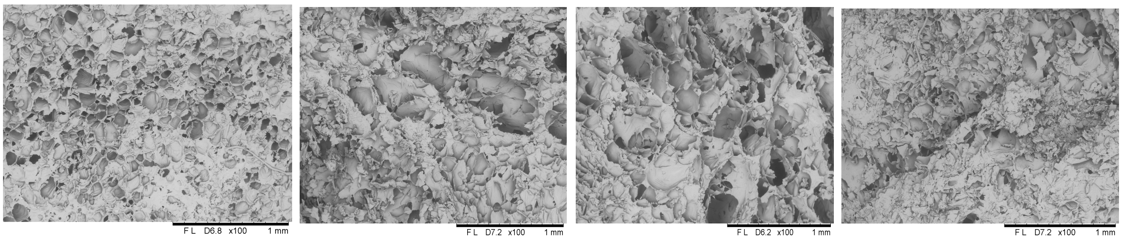

(1) (4) (5) (6)

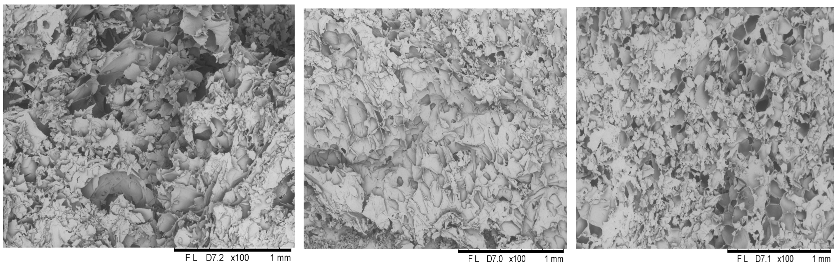

(7) (16) (17)

Figure S1. Beetroot frozen by the quick method. Designation in table 1 (publication, main text).

Combined Freezing

Figure S2 shows the structure of dried red beet subjected to various pre-treatment and frozen with a combined method (8, 9, 10, 11, 18, 19) compared to control sample (2). The control sample (2) was frozen with combined method at -80 ˚C for 2 hours, and then stored at −18 ˚C for 2 weeks. Its structure is characterized by large, closed pores, which confirms low porosity (53.6%) of freeze-dried material (Figure 5 publication, main text). In the visual assessment, samples of blanched red beet (8–11) have larger, partially open pores as opposed to the control sample (2). Increasing the treatment duration affects the changes in structure. A sample of red beet blanched for 1 min (8) is characterized by finer pores, partially closed, and the pore size increases with the duration of blanching. This is confirmed by the results of porosity tests (Figure 5 publication, main text), which showed that extending the treatment time from 1 min to 15 min causes an increase in porosity from 75.26% to 81.5%. Red beet samples blanched longer, but at a lower temperature of 65 °C, are characterized by large pores in the visual assessment. Samples treated with ultrasound (18-19) have a uniform structure with fine pores of similar size.

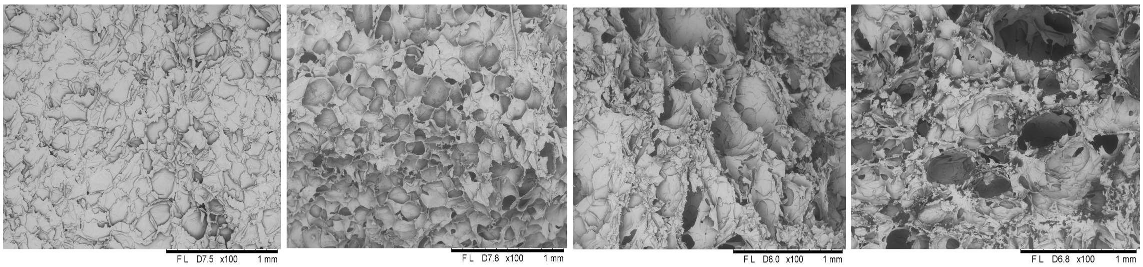

(2) (8) (9) (10)

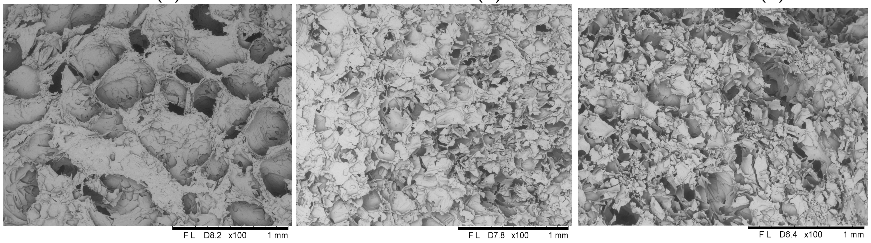

(11) (18) (19)

Figure S2. Beetroot frozen by the combined method. Designation in table 1 (publication, main text).

Slow Freezing

The structure of dried red beet subjected to different pre-treatment and slow-frozen (12, 13, 14, 15, 20, 21) and control sample (3) is presented on Figure S3. The control sample (3) was freeze-dried red beet initially subjected to freezing in water at −18 °C. Its structure was heterogeneous, with partially open pores various sizes. The samples subjected to blanching and frozen slowly (12-15) in the visual assessment were also characterized by pores with different sizes and non-uniform structure. No significant differences were observed between them and the control sample. The results of porosity tests (Figure 6 publication, main text) confirm visual observation of the structure. However, the use of ultrasonic treatment (20-21) caused that the samples were characterized by very small and numerous pores.

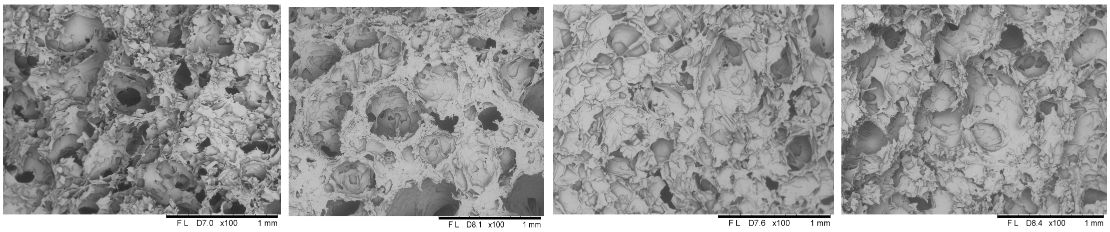

(3) (12) (13) (14)

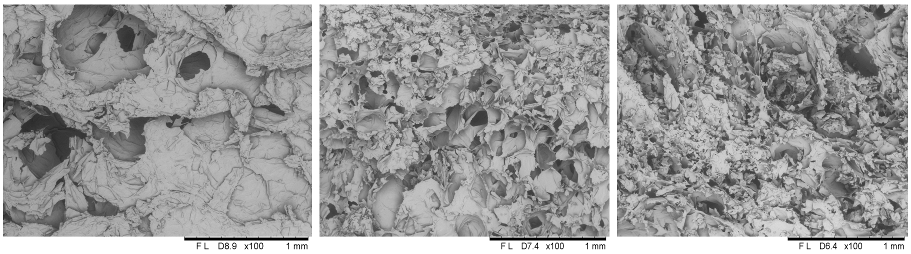

(15) (20) (21)

Figure S3. Beetroot frozen by the slow method. Designation in table 1 (publication, main text).

Depending on the freezing conditions used, changes were noted in the internal structure of the material (Figure S4). The smallest and most numerous pores were obtained in a sample frozen quickly at −80 °C for 2 hours (1). The structure was compact and homogeneous. Cell spaces have similar, regular sizes. Freezing at −80 °C for 2 hours, and then storage for 2 weeks at −18 °C (2) contributed to the formation of numerous closed pores. The freezing method resulted in obtaining the lowest porosity (Figure 14 publication, main text). Freezing at −18 °C for 2 weeks (3) destroyed the structure of dried red beet. The size of the pores present in the visual assessment was varied. Occurring pores were open and large. Visual observations were confirmed by tests of shrinkage (Figure 11 publication, main text) and porosity (Figure 10 publication, main text).

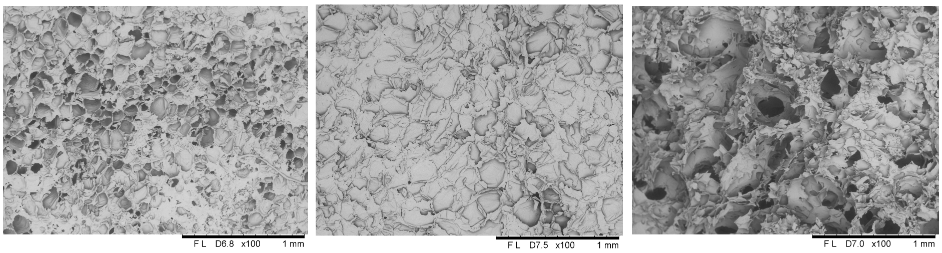

(1) (2) (3)

Figure S4. The effect of freezing conditions on the structure of freeze-dried beetroot. Designation in table 1 (publication, main text).
